# School-level intra-cluster correlation coefficients and autocorrelations for children’s accelerometer-measured physical activity in England by age and gender

**DOI:** 10.1186/s12874-024-02290-7

**Published:** 2024-08-09

**Authors:** Ruth Salway, Russell Jago, Frank de Vocht, Danielle House, Alice Porter, Robert Walker, Ruth Kipping, Christopher G. Owen, Mohammed T. Hudda, Kate Northstone, Esther van Sluijs, International Children’s Accelerometry Database (ICAD) Collaborators, Andrew Atkin, Ulf Ekelund, Dale Esliger, Bjorge H. Hansen, Lauren Sherar, Esther van Sluijs

**Affiliations:** 1 Population Health Sciences, Bristol Medical School, University of Bristol, Bristol, UK; 2 The National Institute for Health Research, Applied Research Collaboration West (NIHR ARC West), University Hospitals Bristol and Weston NHS Foundation Trust, Bristol, UK; 3 NIHR Bristol Biomedical Research Centre, University Hospitals Bristol and Weston NHS Foundation Trust and University of Bristol, Bristol, UK; 4 Population Health Research Institute, St George’s, University of London, London, UK; 5 Department of Population Health, Dasman Diabetes Institute, Kuwait City, Kuwait; 6 MRC Epidemiology Unit, School of Clinical Medicine, University of Cambridge, Cambridge, UK

**Keywords:** Physical activity, Children, Adolescents, Schools, Intra-cluster correlation coefficient, Cluster autocorrelation, Cluster randomised trial, Sample size, ICAD

## Abstract

**Background:**

Randomised, cluster-based study designs in schools are commonly used to evaluate children’s physical activity interventions. Sample size estimation relies on accurate estimation of the intra-cluster correlation coefficient (ICC), but published estimates, especially using accelerometry-measured physical activity, are few and vary depending on physical activity outcome and participant age. Less commonly-used cluster-based designs, such as stepped wedge designs, also need to account for correlations over time, e.g. cluster autocorrelation (CAC) and individual autocorrelation (IAC), but no estimates are currently available. This paper estimates the school-level ICC, CAC and IAC for England children’s accelerometer-measured physical activity outcomes by age group and gender, to inform the design of future school-based cluster trials.

**Methods:**

Data were pooled from seven large English datasets of accelerometer-measured physical activity data between 2002–18 (> 13,500 pupils, 540 primary and secondary schools). Linear mixed effect models estimated ICCs for weekday and whole week for minutes spent in moderate-to-vigorous physical activity (MVPA) and being sedentary for different age groups, stratified by gender. The CAC (1,252 schools) and IAC (34,923 pupils) were estimated by length of follow-up from pooled longitudinal data.

**Results:**

School-level ICCs for weekday MVPA were higher in primary schools (from 0.07 (95% CI: 0.05, 0.10) to 0.08 (95% CI: 0.06, 0.11)) compared to secondary (from 0.04 (95% CI: 0.03, 0.07) to (95% CI: 0.04, 0.10)). Girls’ ICCs were similar for primary and secondary schools, but boys’ were lower in secondary. For all ages, combined the CAC was 0.60 (95% CI: 0.44–0.72), and the IAC was 0.46 (95% CI: 0.42–0.49), irrespective of follow-up time. Estimates were higher for MVPA vs sedentary time, and for weekdays vs the whole week.

**Conclusions:**

Adequately powered studies are important to evidence effective physical activity strategies. Our estimates of the ICC, CAC and IAC may be used to plan future school-based physical activity evaluations and were fairly consistent across a range of ages and settings, suggesting that results may be applied to other high income countries with similar school physical activity provision. It is important to use estimates appropriate to the study design, and that match the intended study population as closely as possible.

**Supplementary Information:**

The online version contains supplementary material available at 10.1186/s12874-024-02290-7.

## Background

Evaluating the effectiveness of public health interventions is important to inform and develop policy recommendations. Randomised controlled trials (RCTs) are often used to evaluate public health interventions as they tend to be less susceptible to bias than other designs [1]. Often individuals fall naturally into groups (for example schools, workplaces, or geographical locations), or clusters, and it is important to take such clustering into account as individuals within the same cluster are often more similar to each other than to individuals in other clusters [2]. Cluster-based designs, such as cluster RCTs, cluster cross-over trials and stepped wedge designs, are studies in which clusters are randomised rather than the individuals within these groups [2]. Such designs are common in public health settings, where the intervention itself may also be implemented at the cluster level, for example a school programme to increase children’s physical activity, and thus not allow for individual-level randomisation. However, the design and analysis of cluster-based studies is more complex, with larger sample sizes required than for an individual randomised study. The larger the intra-cluster correlation coefficient (ICC), the larger the sample size required, and ignoring the clustering in the design can result in an underpowered study [2].

There is a wide literature concerning sample size estimation for cluster-based designs [2–6], although much is based in a clinical rather than public health setting. Approximate formulae are commonly provided in the form of an adjustment to the sample size required for an individually-randomised trial, and rely on the ICC, which captures the amount of variance explained at the group level [7, 8]. Depending on the study design, there may also be other correlations required for accurate sample size estimation. For example, studies with multiple measures over time in the same clusters, such as stepped wedge designs or cluster RCTs with baseline measurements, may need to take into account cluster autocorrelation (CAC) [9] which measures similarity in the outcome in the same cluster over time. In addition, cohort cluster designs with repeated measurements on the same individuals, should also account for individual autocorrelation (IAC) [4, 5], which captures similarity in outcome measures for the same individual. Accounting for these correlations is important for the same reason as accounting for clustering, as they all capture different ways in which measurements are correlated and thus can affect the required sample size. Note that the ICC, CAC and IAC are features of the clusters or individuals themselves and the outcome measure, and not under the control of the researcher. Reviews that summarise data on ICCs drawn from multiple datasets show considerable variability, with estimates between 0 and 0.2 depending on the outcome, type of cluster, country and population [10–16]. In particular, ICCs are typically larger for smaller cluster sizes, for example, varying between a median of 0.002 at district health level to 0.02 at postcode sector level [12]. Far less is known about the CAC, and the few estimates that have been published range between 0.3 and 0.9 with wide confidence intervals for a mix of clinical and risk factor outcomes, and so differences by outcome or cluster type are not clear [15, 16]. Finally an estimate of the IAC is also needed whenever the same individuals are measured at multiple time points; as this is an individual rather than cluster-level correlation, estimates are generally easier to derive from other studies of both cluster and individual trials. Accurate and precise estimation of the ICC and CAC, and the IAC for cohort designs, is therefore important in planning a study to ensure the sample size is sufficient to detect an intervention effect. However, pilot studies are typically too small to provide robust estimates [17], with a recent review of school-based ICCs finding very little correlation between ICCs estimated from a pilot and the full trial [18]. Therefore, pilot studies are generally not suitable for estimating ICCs or CACs, although can provide other data needed for sample size estimation and study design, including the IAC.

Cluster-based designs are particularly well-suited to evaluate children’s physical activity interventions, as schools form obvious clusters and make good targets for intervention. Estimates of ICCs for children’s physical activity vary, depending on the outcome used. Physical activity guidelines in the UK, and in many other countries, are for children to achieve an average of at least 60 min of moderate to vigorous physical activity (MVPA) per day [19, 20], and so whole-day MVPA is a common primary outcome in such studies. ICCs for MVPA are between 0.01–0.06 for self-reported physical activity [13, 21–24] and higher for accelerometer-measured physical activity at 0.06–0.13 [25–28]. Estimates of average sedentary time across the whole day are less often reported, but ICCs tend to be lower, at 0.02 for self-reported sedentary time [13] and 0.06–0.09 for accelerometer-measured sedentary time [29, 30]. These ICC estimates for both MVPA and sedentary time are often calculated using relatively few schools (< 30) and usually reported without confidence intervals. Unlike other health-related behavioural outcomes (such as smoking and alcohol), where much of the cluster dependence is due to demographic similarities within schools, this is not the case for physical activity, where MVPA ICCs adjusted for individual covariates remain similar to unadjusted estimates [13, 27]. As schools play a specific role in facilitating physical activity, with a requirement to provide a certain amount of activity within the school curriculum, there is a direct mechanism for similarities between children in the same school. This is reflected in variation in ICCs of MVPA throughout the day, for example, 0.25 in school and 0.06 after school [28], and differences between weekdays (0.13) and weekends (0.05) [27]. As school size, facilities, curriculum and structure of the school day will all impact on the ICC, we might also expect ICCs to be country-specific, and differ by type of school, and especially by age. For example, in the UK, primary schools (ages 4–11) are typically smaller in terms of pupil numbers and physical size, have fewer specialist sports facilities and have a single class teacher rather than multiple subject teachers, compared to secondary schools (ages 11–18). While reported ICCs for children’s MVPA tend to be higher than for adolescents [26], the majority of published estimates are for adolescents and so it is not clear if and how they differ. We are also not aware of any studies that compare ICCs between boys and girls in the same schools. As girls consistently engage in less physical activity than boys on average [31], this may be an important difference, for example if girls are more reliant on the school environment, especially as interventions are often specifically designed to target girls.

The aim of this paper is to estimate the ICC, CAC and IAC for UK children’s accelerometer-measured weekday and whole week MVPA and sedentary time by age group and where possible, gender, to help inform the design of future school-based cluster trials of physical activity interventions. We use secondary data from seven large observational studies of accelerometer physical activity data, where participants are clustered within schools and that include a sufficiently large number of schools for reliable estimates. We pool data to estimate ICCs for different age groups, stratified by gender, and compare these to ICCs for children’s self-report physical activity. We also estimate the CAC and IAC from those datasets that include longitudinal data. To the best of our knowledge, there is no literature that has explored whether there are differences in ICC by accelerometer type or processing protocols e.g. cut-points, epochs or definition and number of valid days, and so a secondary aim is to explore the effect of different accelerometer processing criteria on the ICCs. Finally, we will illustrate how these findings affect estimates of the required sample size in a worked example.

## Methods

### Data

This paper uses secondary data from seven English studies of children’s accelerometer-measured physical activity. We began by selecting studies which included data on school membership from the International Children’s Accelerometry Database (ICAD) [32], which pooled and harmonised accelerometer physical activity data on children and adolescents. We restricted to datasets from England as ICCs are likely to differ between countries due to differences in school structure, facilities and curriculum requirements [33]. We then identified additional studies from within the University of Bristol and from the literature which also used Actigraph waist-worn accelerometers, were based in England and contained data from at least 20 schools. The seven included studies were the Avon Longitudinal Study of Parents and Children (ALSPAC) [34, 35]; Sport, Physical activity and Eating behaviour: Environmental Determinants in Young people (SPEEDY) [36] and Personal and Environmental Associations with Children's Health (PEACH) [37] studies from ICAD, and the B-Proact1v study [38, 39], Action 3:30 feasibility trial (A3:30) [40], Active for Life Year 5 (AFLY5) trial [41, 42] and the Child Heart And health Study in England (CHASE) [43]. All studies received ethical approval and informed consent [44] (see individual studies for further details). The included studies consisted of four cohort studies, one cross-sectional study and two cluster RCT trials (Table 1). For the cluster RCT trials, we used baseline data from both arms and follow-up data from the control arm only. Data covered ages 5 to 16 years, comprising over 13,500 pupils from 540 schools, with 60% from primary schools and 40% from secondary schools.

**Table 1:** Summary of datasets and which parameters can be estimated

Dataset	Study type	Region	Age range	No. schools	No. pupils	Estimates^a^
Accelerometer-measured						
Action 3:30	cRCT Trial	SW England	9–11	20	529	ICC, [CAC, IAC]
AFLY5	cRCT Trial	SW England	8–10	60	2028	ICC, [CAC, IAC]
ALSPAC	Cohort	SW England	11–16	221	3766	ICC, CAC, IAC
B-Proact1v	Cohort	SW England	5–11	57	2054	ICC, CAC, IAC
CHASE	Cross-sectional	England	9–10	77	2040	ICC
PEACH	Cohort	SW England	10–12	23	1255	ICC, IAC
SPEEDY	Cohort	E England	9–14	92	2009	ICC, CAC, IAC
Self-report						
ALCYP	Cross-sectional	England	5–16	1815	107,922	ICC

*cRCT* cluster randomized controlled trial, *ICC* intra-cluster correlation coefficient, *CAC* Cluster Autocorrelation, *IAC* Individual Autocorrelation, *E* East, *SW* southwest

^a^square brackets indicate quantities estimable from control arm only and thus with smaller sample size

All studies used hip-worn Actigraph accelerometers (Table S1). Data from the ICAD studies were harmonised to use 60s epochs, Evenson cut-points (with MVPA defined as < 2295 counts per minute (cpm)), and a valid day defined as a minimum of 480 min of wear time. Data from the B-Proact1v study was re-processed to the same criteria for comparability, while the other studies used smaller epochs of 5-10s. All but one study used Evenson cut-points and similar wear time of 480–500 min, with the remaining study using a lower MVPA threshold of 2000 cpm and a longer valid day of at least 600 min of wear time (Table S1). Note that the impact of different processing criteria is assessed in the Analysis section.

From each study, we extracted accelerometer data (mean minutes of MVPA, sedentary time and wear time for weekdays and the whole week), and demographics (age and gender) for each time point for each child. Age was grouped into categories based on England school year: Year 1 & 4 (age 5–6 and age 8–9; note that no studies provided data on children between ages 6 and 8), Year 5 (age 9–10), Year 6 (age 10–11), Year 7 (age 11–12), Year 8 & 9 (age 12–14) and Year 10 & 11 (age 14–16). Primary schools in England comprise Years 1–6, and secondary schools cover Years 7–11.

To compare ICCs for accelerometer-measures and self-report physical activity, we also used data from the Active Lives Children and Young People survey 2018–19 (ALCYP) [45, 46]. This is an annual cross-sectional survey commissioned by Sport England, which collects self-report physical activity data for children aged 7–16 from a large representative sample (*n* = 107,922 across 1815 schools) across the whole of England to inform UK government strategy. Children were asked detailed questions about participation and intensity in a range of activities both in and out of school on each day of the previous week. We excluded children aged 5–7 years where physical activity data were not comparable as the data were reported by parents rather than the children, and used the provided school year groups: Year 3 & 4 (age 7–9) Year 5 & 6 (age 9–11), Year 7 & 8 (age 11–13) and Year 9 & 11 (age 13–16). Weekday and whole week MVPA was calculated from the provided derived variables of minutes of MVPA by day.

### Analysis

The primary outcome was mean weekday MVPA, with secondary outcomes of mean MVPA across the whole week, sedentary time for weekdays and the whole week. We restricted analysis to those pupils who provided at least two days of valid data. Physical activity outcomes and gender were summarised by school year group. All analysis was run in Stata v17 [47], and sample size estimates were calculated using the clusterpower package in R.

#### Intra-cluster correlation coefficients

To produce model-based estimates of the ICC by school year group, we used linear mixed effect models to take into account variability across studies, with separate models for primary and secondary schools, due to differences in total variability. Three-level mixed models included fixed terms for age and accelerometer wear time, and random effect terms for study, individual repeated measures and school-level random coefficients for each school year group. ICCs were calculated separately for each school year group from the total and between-school variance estimates, with approximate 95% confidence intervals obtained using the delta method [48] on the logit scale and back-transformed to give a 95% confidence interval for each ICC. We also ran subgroup analyses by gender. Models were run for weekday and whole week MVPA, and for weekday and whole week sedentary time. For longitudinal data, we also estimated ICCs for weekday MVPA adjusted for baseline (i.e. previous MVPA measurement), for primary and secondary schools. Due to smaller sample sizes we did not break down further by school year group, or stratify by gender. Finally, we ran models for weekday and whole week self-report MVPA using the ALCYP data (with no study random effect) using a linear model. We chose not to transform the data, despite being heavily right-skewed, as this is not often done in practice and so ICCs on the transformed scale would be less useful, and potentially misinterpreted.

#### Effect of accelerometer processing

To explore whether ICCs differ depending on the accelerometer processing, we ran a series of additional analyses. We used the B-Proact1v data from Year 6 only, where we had access to the raw accelerometer data and so could re-process in different ways, to look at differences in the cut points to determine MVPA (2000, 2295), resolution (5s 10s and 60s epochs), definition of a valid day (minimum wear time required for inclusion: 400 min, 480 min and 600 min), and the minimum number of valid days for inclusion (one, two or three days).

#### Cluster and individual autocorrelations

As the autocorrelations CAC and IAC may depend on the follow-up time between measurements, we used empirical estimates from the Pearson correlation coefficients for each study/follow-up time combination. Where studies covered the transition between primary and secondary school, we included only data within the same school for estimating the CAC, but used all measurements across multiple schools for the IAC. These were transformed using Fisher transformations for approximate normality, combined using meta-analysis and then back-transformed to produce joint estimates of the CAC and IAC by follow-up time. Due to the smaller numbers of pupils and schools available to calculate the autocorrelations, we did not separate by school type or gender. We also excluded schools with fewer than five pupils in calculating the school-level correlations for the CAC.

#### Example sample size calculations

To illustrate how the estimates produced in earlier sections can be used to estimate sample sizes in future cluster-based evaluations of children’s physical activity interventions, we estimated the number of schools required to detect an effect size of a 5 min increase in weekday MVPA (a difference likely to have a meaningful health impact [49]), with power 80% and significance level 5%, for six different study designs (cluster RCT, cluster RCT adjusted for baseline, cross-sectional and cohort stepped wedge designs with two and three steps). We calculated sample sizes for Year 5 (primary) and Year 10/11 (secondary) separately, using estimates of the total variation, ICC, CAC and IAC from the analysis above. We used two values of the ICC, the point estimate and the upper bound of the 95% confidence interval, to investigate how sensitive the resulting sample sizes were to different values of ICC. We assumed an achieved sample size of 25 pupils per school for primary and 50 for secondary; these are smaller than typical year groups to allow for nonresponse. We also explored different values of ICC and varying the number of pupils per school.

## Results

The sample comprised 13,650 children from 540 schools across seven studies, measured at a total of 19 time points (Table 2). Around half the pupils (53%) were female, with 60% of the measurements taken in primary schools and 40% in secondary schools. Missing accelerometer data was around 4–10% for most studies (with a higher missingness rate of 20% for weekdays in the AFLY5 study) resulting in 21,076 valid weekday measurements and 21,649 valid measurements across the whole week (Table S2).

**Table 2:** Sample characteristics summarised across all datasets

	Accelerometer		Self-report	
	% or mean	N or (sd)	% or mean	N or (sd)
**Child characteristics**	***N* = 13,650**		***N* = 107,922**	
% female	53%	7211	52%	52,719
**Year group**	***N* = 23,749**		***N* = 107,922**	
Year 1 & 4 (Age 5–9)	18%	4211	20%	23,012
Year 5 (Age 9–10)	23%	5364	24%	26,989
Year 6 (Age 10–11)	19%	4622		
Year 7 (Age 11–12)	14%	3297	24%	27,227
Year 8 & 9 (Age 12–14)	17%	3919		
Year 10 & 11 (age 15–16)	10%	2336	28%	32,020
**Primary**	***N* = 14,197**		***N* = 107,922**	
Weekday MVPA (min): Mean (sd)	55.8	(24.8)	86.3	(89.4)
Overall MVPA (min): Mean (sd)	54.3	(24.0)	78.4	(81.9)
Weekday sedentary (min): Mean (sd)	418.3	(117.6)		
Overall sedentary (min): Mean (sd)	406.4	(116.0)		
**Secondary**	***N* = 9552**		***N* = 107,922**	
Weekday MVPA (min): Mean (sd)	54.7	(28.1)	84.7	(86.2)
Overall MVPA (min): Mean (sd)	50.9	(26.1)	75.1	(75.7)
Weekday sedentary (min): Mean (sd)	425.0	(96.0)		
Overall sedentary (min): Mean (sd)	412.3	(91.0)		

Child characteristics are summarised by child; year group, primary and secondary are summarised for all measurements at that time point, and may include multiple measures per child

*MVPA* moderate-to-vigorous physical activity, *sd* standard deviation

### Intra-cluster correlation coefficients

**Fig. 1:**
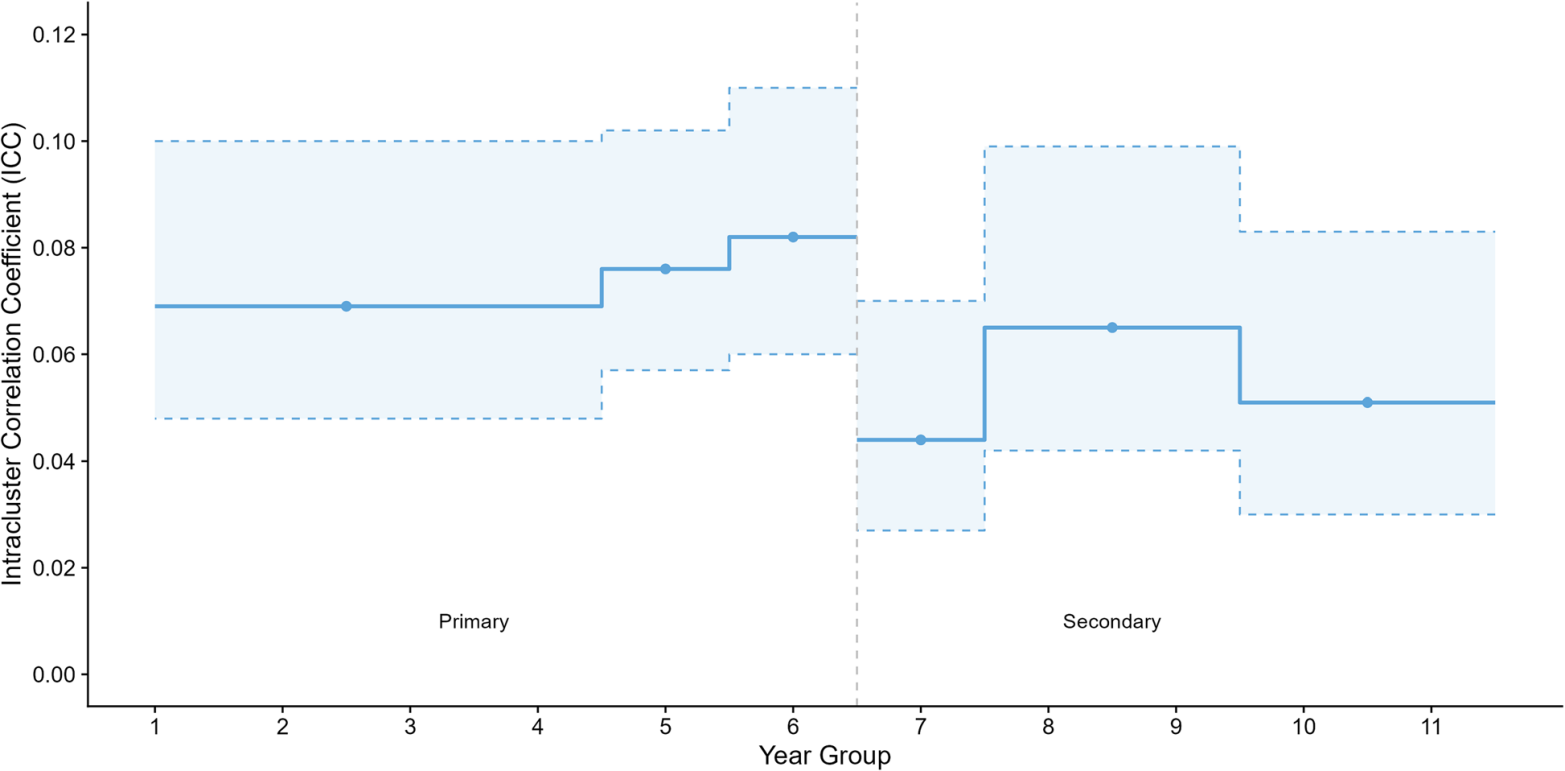
Estimated intra-cluster correlation coefficient (ICC) and 95% confidence intervals by age group

Modelled ICCs for weekday accelerometer MPVA are shown in Fig. 1, with full estimates and 95% confidence intervals given in Table 3. ICCs were slightly higher for children in primary schools (between 0.07 (95% CI: 0.05, 0.10) for Years 1 & 4, and 0.08 (95% CI: 0.06 – 0.11) in Year 6) compared to secondary (between 0.04 (95% CI: 0.03, 0.07) for Year 7, and 0.07 (95% CI: 0.04 – 0.1) in Years 8 & 9). There were only small differences by school year group, with ICCs increasing slightly in primary school with age, and highest in secondary school at Year 8/9. ICCs for boys and girls (Figure S1; Tables S3 & S4) were similar in primary schools, but in secondary schools ICCs were higher for girls than for boys (a maximum of 0.10 (95% CI: 0.06, 0.15) for girls, and 0.04 (95% CI: 0.02 – 0.08) for boys). ICCs for whole week MVPA were slightly lower than weekdays only. ICCs for sedentary time were lower than for MVPA, with estimates of between 0.03 (Years 1 & 4; 95% CI: 0.01, 0.06) and 0.05 (Year 6; 95% CI: 0.03 – 0.11) in primary schools and < 0.01 in secondary schools. ICCs for sedentary time did not differ notably by gender. However, total within-study variation was larger in secondary schools vs primary schools and in boys vs girls for all physical activity outcomes (Table S5).

**Table 3:** Intra-cluster Correlation Coefficients (ICCs) for MVPA and sedentary time by school year group

	Weekday		Whole week	
	ICC	95% CI	ICC	95% CI
MVPA				
Year 1 & 4	0.069	(0.048, 0.100)	0.065	(0.044, 0.093)
Year 5	0.076	(0.057, 0.102)	0.056	(0.040, 0.077)
Year 6	0.082	(0.060, 0.110)	0.071	(0.052, 0.097)
Year 7	0.044	(0.027, 0.070)	0.028	(0.016, 0.049)
Year 8 & 9	0.065	(0.042, 0.099)	0.048	(0.030, 0.076)
Year 10 & 11	0.051	(0.030, 0.083)	0.041	(0.024, 0.069)
Sedentary time				
Year 1 & 4	0.025	(0.011, 0.055)	0.021	(0.009, 0.046)
Year 5	0.033	(0.015, 0.068)	0.025	(0.011, 0.052)
Year 6	0.053	(0.025, 0.106)	0.041	(0.019, 0.084)
Year 7	0.007	(0.003, 0.021)	0.007	(0.002, 0.019)
Year 8 & 9	0.008	(0.003, 0.022)	0.004	(0.001, 0.015)
Year 10 & 11	0.007	(0.002, 0.025)	0.005	(0.001, 0.022)

*CI* confidence interval, *MVPA* moderate to vigorous physical activity

ICCs adjusted for baseline (that is, the previous available measurement; Table S6) were slightly smaller (0.06 for primary; 0.04 for secondary) than unadjusted ICCs but showed similar patterns, with higher ICCs for primary schools compared to secondary, for weekdays compared to the whole week and for MVPA compared to sedentary time. The total variation was lower in models adjusted for baseline (Table S5). Self-report MVPA was highly skewed, with ICCs similar to those for accelerometer-measured MVPA, but no substantial differences between girls and boys or between weekday and whole week estimates (Table S7).

### Effect of accelerometer processing

Looking at the BProact1v Year 6 data only, the choice of MVPA cut-point, resolution, definition of a valid day and minimum number of valid days made only minor differences to the ICCs (Table S8). All ICCs were estimated between 0.127 and 0.147, compared to 0.137 for data processed to the criteria used in the main analysis. The largest differences were for a lower MVPA threshold of 2000 (slightly higher ICC of 0.147) and for a longer valid day of 10h (slightly lower ICC of 0.127).

### Cluster and individual autocorrelations

**Fig. 2:**
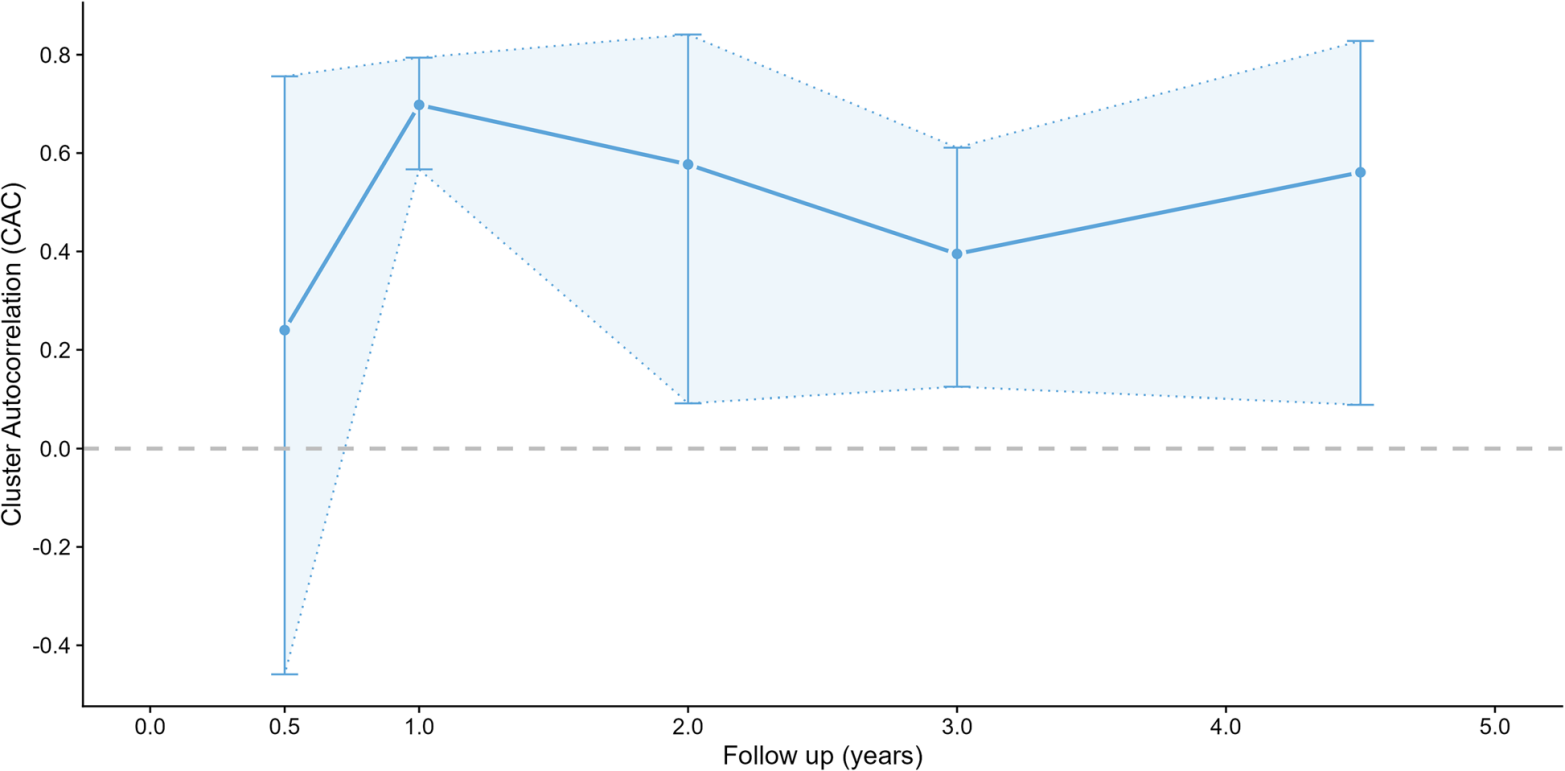
Cluster autocorrelation (CAC) by length of follow-up

**Fig. 3:**
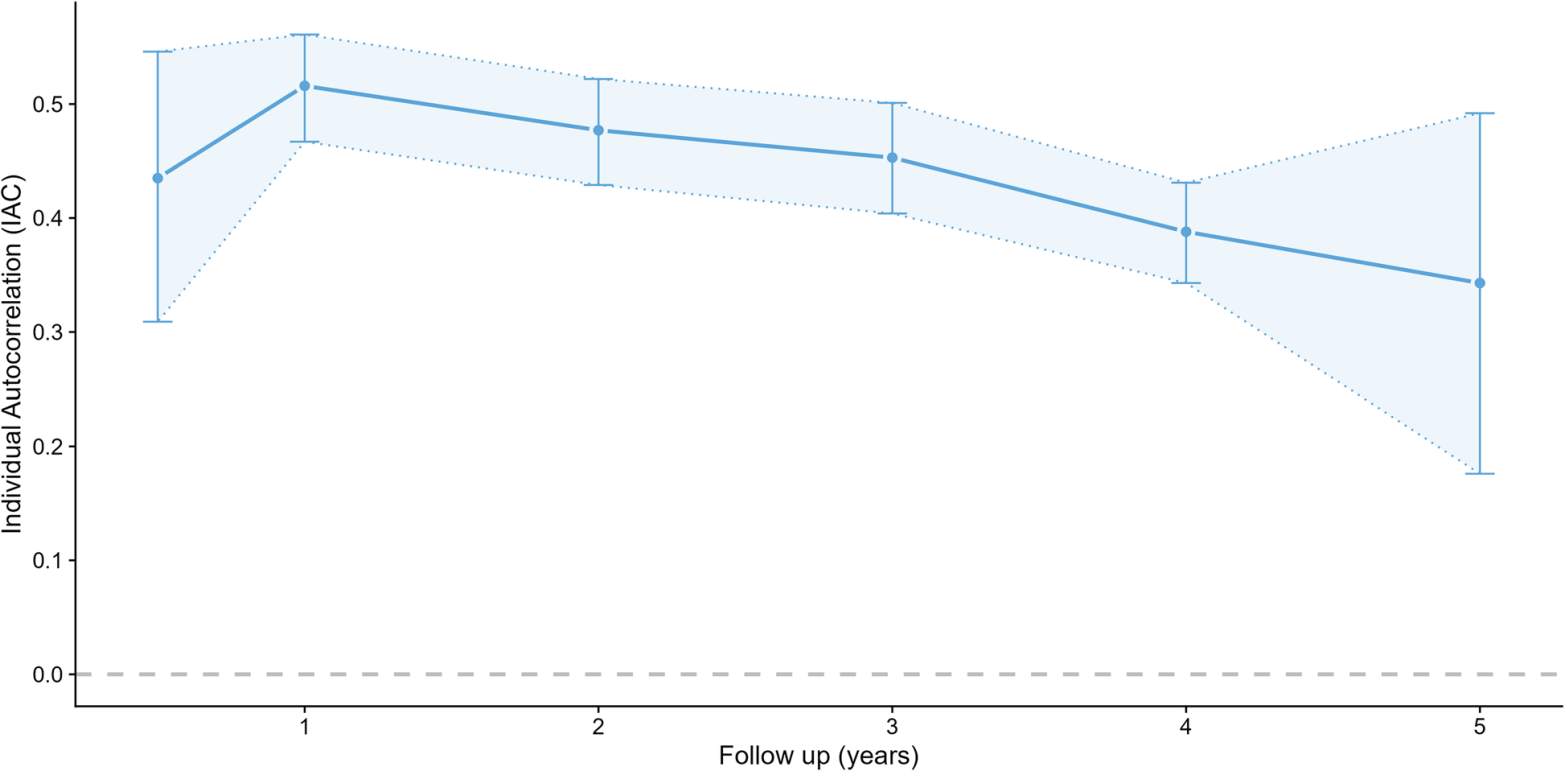
Individual autocorrelation (IAC) by length of follow-up

The CAC (Fig. 2; Table S9) ranged between 0.24 and 0.70 with no consistent pattern over time, and wide confidence intervals, due to relatively small numbers of schools. The estimated CAC for weekday MVPA follow-up times of 1–5 years was 0.60 (95% CI: 0.44—0.72). The CAC for follow-up of less than a year was around half the size, but is based on only one study comprising 10 schools. CACs for sedentary time were slightly lower. The individual autocorrelations for MVPA (Fig. 3; Table S10) showed a possible slow decrease over time, from 0.52 after one year, to 0.34 after 5 years, but confidence intervals were wide. IACs for sedentary time were slightly lower, but showed a similar decreasing pattern with length of follow-up. There were no differences in IAC between weekday and the whole week.

### Example sample size calculations

Table 4 and Tables S11 & S12 estimate the number of schools required to detect an effect size of a 5 min increase in weekday MVPA for a future cluster-based evaluation of children’s physical activity interventions, with power 80% and significance level 5%, and using estimates of the total variation, ICC, CAC and IAC from the results presented above. We considered six different study designs: cluster RCT, cluster RCT adjusted for baseline, cross-sectional and cohort stepped wedge designs with two and three steps. Table 4 shows that the achieved number of schools required would range substantially from 33–94 for primary and 24–96 for secondary schools, depending on the cluster design used, and assumptions about the ICC and autocorrelations. Although the ICC in secondary schools is lower, more secondary schools are needed than primary for the same effect size and power, due to the higher total variability. The number of schools required increased notably if the upper bound of the ICC 95% confidence interval was used rather than the point estimate, with 6 extra schools required for the cluster RCT in primary schools, and 14 additional secondary schools required for the same power. The simplest design, the two-arm cluster RCT, required the most schools (68–96 depending on parametrisation). Repeated measures, either in schools or pupils, increased the power and reduced the required sample size. For example, the cross-sectional two-step stepped wedge and cluster RCT adjusted for baseline both reduced the number of schools required by around 30% (from 82 to 54–62 schools in primary and from 68 to 46–50 schools in secondary). The cohort two-step stepped wedge and cross-sectional three-step stepped wedge reduced by around half (to 39–48 primary schools and 30–40 secondary schools) and the largest reductions were for the cohort three-step stepped wedge by nearly two thirds (30 primary schools, 24 secondary schools). Increasing the number of pupils per school reduced the sample size to a lesser degree with up to 20–30% reduction for an increase of 25 in number of pupils (Table S11 & S12), but this had less effect for larger ICCs and as pupils numbers increased beyond 50. The larger reductions were in the stepped wedge designs, which exploit correlation between pupils at different time points.

**Table 4:** Number of schools required to detect an increase of 5min weekday MVPA for different designs

	Primary (Y5) 25 pupils per school		Secondary (Y10/11) 50 pupils per school	
	Point estimate	Upper bound	Point estimate	Upper bound
**Cluster RCT**				
Two-arm^a^	82	94	68	96
adjusted for baseline^b^	54	70	46	60
**Stepped wedge (same schools): 2 steps**				
cross-sectional^a^: CAC = 0.6	62	68	50	64
Cohort^a^: CAC = 0.6, IAC = 0.5	48	54	40	54
**Stepped wedge (same schools): 3 steps**				
cross-sectional^a^: CAC = 0.6	39	42	30	39
Cohort^a^: CAC = 0.6, IAC = 0.5	30	33	24	33

*MVPA* moderate-to-vigorous physical activity, *RCT* randomised controlled trial, *ICC* intra-cluster correlation coefficient, *CAC* cluster autocorrelation, *IAC* individual autocorrelation

Assuming 80% power; 5% significance level; total standard deviation: primary = 23.2 min, secondary = 27.5 min

^a^ICC point estimate (upper bound): primary 0.08 (0.10) secondary 0.05 (0.08)

^b^ICC point estimate (upper bound): primary 0.06 (0.09), secondary 0.04 (0.06)

## Discussion

In this paper we have combined data from over 13,500 pupils from 540 schools to provide a comprehensive set of estimates of school-level ICCs and autocorrelations for accelerometer-measured physical activity in children and adolescents which can be used to plan new school-based cluster trials of physical activity interventions in schools. The ICC estimates for weekday MVPA are broadly consistent with other accelerometer-measured estimates reported in the literature, which range between 0.07 to 0.13 [25–27], although these studies all involved fewer schools and estimates were presented without measures of precision. ICCs were slightly higher in primary schools, compared to secondary schools, as seen elsewhere [26], with only small differences by age within school type. Due to using secondary data sources, we looked at physical activity summaries across the whole day only, although other studies suggest that ICC estimates for activity during school hours only are likely to be much higher [28]. The specific criteria used in accelerometer processing had very little effect on the ICC, and we saw no differences by MVPA cut-points, resolution, definition of a valid day or number of days included. While these factors can affect estimates of minutes of MVPA themselves, the ICC is a relative measure that captures similarity between measurements and is thus less influenced by these choices as all individual measurements within a school are higher or lower in the same way. We were unable to explore the impact of wear and non-wear definitions, which tend to affect sedentary time in particular, but it seems reasonable to assume that such definitions will similarly have less impact on ICC estimates than on physical activity estimates themselves. All estimates of ICCs were between 0.04–0.08 with overlapping confidence intervals, suggesting that accelerometer-measured ICCs are fairly consistency across a range of ages and settings. This therefore suggests that the results presented here may be useful more widely, for example in other UK nations and other high income countries that have similar physical activity provision within schools. In such cases, we suggest that in the absence of more specific estimates, an ICC of 0.10 for weekday MVPA could be used for sample size calculations, based on the upper 95% CI bounds to reduce the risk of underpowering a study. Corresponding estimates for sedentary time are thus 0.05 -0.06 for ages 6–11 (English primary schools) and 0.02–0.03 for ages 11–16 (English secondary schools).

When considering boys and girls separately, in primary schools, both genders had similar ICCs for weekday MVPA of 0.08–0.12. Note that these ICCs are a little higher than the estimate of 0.07–0.08 for boys and girls combined. This apparently counter-intuitive result is because ICCs are relative measures that depend on the total variation denominator, which differs between genders, with higher individual variability for boys than for girls. In secondary schools, ICCs were lower for boys, but for girls they remained at similar levels to primary school. Girls typically engage in less physical activity than boys especially in adolescence [31], and so the ICC will be higher as the total variation is lower in a less active population. These results highlight the importance of using ICCs that are closest to the intended population in planning a study, especially when considering subgroups, where ICCs for combined groups may differ due to differences in total variation. For example, a study involving Year 7 girls only using the ICC for girls would require 60 schools for 80% power. However, if an ICC of 0.04 based on boys and girls together is used, the required number of schools is estimated at only 42, which would result in an underpowered study at 65% power.

It is always important that studies are adequately powered, but in public health underpowered studies may risk missing interventions that have relatively small individual effects, but have the potential for much greater impact at population level. Our examples of sample size calculation suggest that relatively small differences of 0.02 in the ICC can increase the number of schools required by around 20% (corresponding to an increase of 14–18 for a two-arm cluster RCT for an effect size of 5 min of MVPA). This is particularly an issue for school-based trials of physical activity interventions, as recruiting the additional schools this requires is resource-intensive. Developing interventions in this area is very important as we know that children’s physical activity patterns have changed since the COVID-19 pandemic [50–53], but it is equally important that such interventions are properly evaluated. The sensitivity of school sample sizes to relatively small differences in the ICC means that considering a plausible range of ICCs can lead to much larger studies than in other settings, which may have funding implications for the amount and availability of sufficient funding to test interventions at the necessary scale. While an increase in ICC can be offset to some extent by increasing the number of pupils recruited per school, the eligible population is restricted, especially in primary schools, and increases in power are limited after around 50 pupils per school. However, using ICCs based on the wrong age group, population or from non-physical activity outcomes can also potentially result in an under-powered study. For example, from our estimates, a primary school study of Y5 pupils should use an ICC of 0.08 for weekday MVPA, with 82 schools required for a two-arm cluster RCT with 80% power. If, instead, an ICC of 0.04 is used, based on secondary schools MVPA across the whole week, the calculated sample size is 56 schools, with true power 64%. Using ICC estimates of self-reported physical activity outcomes from the literature [13, 21, 22] of around 0.02 will reduce this even further to 44 schools and 53% power. The adequate powering of studies is important in terms of moving the evidence for effective physical activity strategies in children and adolescences forward [33], and so we recommend that a range of plausible ICC values should be explored, to assess the sensitivity of the estimated sample sizes to different values, such as using the upper 95% bound as well as the point estimate, as in our example.

There are a number of observations that we can make with respect to the efficient design of cluster-based trials of physical activity outcomes in children and adolescence. Designs which involve repeated measurements increase the power of the study, or reduce the number of schools required. Note that when designing a cluster RCT adjusted for baseline, it is important to use both ICC and total variation estimates that have also been adjusted for baseline, and studies that report ICCs should be clear about whether any adjustment has been made. While the ICCs adjusted for baseline are only slightly smaller than unadjusted ICCs in our results, repeated measurements on the same children reduce the total variation and so fewer schools are needed for a study with the same power. This study design implicitly takes account of the autocorrelation between successive measurements, and is related to including the CAC and IAC in the stepped wedge design [5]. An advantage of the stepped wedge design is that it can be used for both cross-sectional and cohort samples as it does not require the same pupils to be measured [4]. However, it is more complex to design, run and analyse, and while the cohort design has substantial benefits in terms of required sample sizes, the numbers presented in Table 4 assume that repeated measurements at baseline and each step are achieved for all pupils. In practice, repeated measures may result in lower pupil numbers, especially as the number of steps increases. Accurate estimates of power for the stepped wedge design should also account for the CAC and IAC, with the former particularly important, as some simplified models for sample size estimation assume a CAC of 1 [54], which will result in under-powering of the study [4]. While we were able to produce reasonable estimates for the IAC, the estimate of the CAC was still not precise even when pooling schools from multiple studies. Models and sample size formulae have been developed to consider CAC correlation structures that decay over time [55]. Unfortunately, our analysis was unable to provide accurate or precise estimates of the CAC under a year, due to limited data and seasonality in physical activity outcomes. Our results indicate that after one year, any further decay is weak, with the CAC levelling at around 0.5–0.6, reflecting that schools are more consistent over time than individuals. While we are unable to provide reliable estimates for shorter-term CACs, we suggest that a range of plausible values are considered at the design stage. However, care should be taken in using complex correlation structures in sample size calculations for evaluating children’s physical activity (although such models may be useful in analysis) as this might artificially increase power due to overconfidence in estimates.

The primary focus of this paper is on estimating ICCs for use in sample size estimation. However, unlike clinical or primary care settings, where clustering is often a nuisance factor related to the data collection process, in physical activity and many other public health contexts, the clustering is a direct feature of physical activity and even the intervention itself. As a measure of clustering, the ICC tells us about the extent to which schools (and/or classes), and school-related factors, explain between-child variation in physical activity. Thus school factors, such as playground equipment, active travel, PE provision and active after-school clubs [56, 57], play more of a role in primary schools than in secondary schools. Similarly, the higher ICC for girls in secondary school may reflect that girls do comparatively little physical activity outside the school context and so the influence of schools is greater, even though they typically engage in less physical activity than boys. This makes schools potentially good targets for developing interventions, although it is worth noting that the largest source of variation between children is still due to individual factors [57]. Unfortunately we were unable to separate school-level and class-level variability, and so the reported ICCs combine both sources of variation; it is likely that a substantial proportion of the observed school-level clustering is at the class level. Understanding different levels of variation can help determine whether interventions should be focused on group or individual level, similar to applications in occupational health [58]. However, it also makes interventions and their evaluation more complex, and instead of just estimating ICCs we need to properly understand when and how clustering affects physical activity outcomes, especially when dealing with a complex intervention that may affect the ICC itself.

### Strengths and limitations

This paper has a number of strengths. We have combined data on over 500 schools, which has allowed more precise estimates of ICCs and autocorrelations than have been previously reported. Combining data from seven different studies covering a wide age range of ages 6–16 years, and has enabled us to explore patterns by age and gender. The mixed effects model used to estimate the ICCs is consistent with that used for sample size formulae and analysis of the various cluster-based designs considered. We were also able to produce estimates of the autocorrelations, CAC and IAC. However, estimates of autocorrelation were limited in their ability to explore short-term follow-ups of less than a year, and in particular estimates of the CAC were still imprecise despite the large sample sizes. Although the studies covered a wide age range, each age group was often dominated by a single study, which means that observed patterns by age may be related to differences in studies rather than school year group. As this was a secondary analysis, we do not have any information on missing data beyond no value being provided in the dataset. Accelerometer processing criteria differed between studies, in terms of wear time definitions, criteria for valid days and number of days included in the study, which may affect results, although our limited sensitivity analysis suggests that unlike physical activity estimates themselves, ICC estimates are reasonably robust to these differences. We were also unable to look at ICCs for during school hours only, or to explore class-level variability. Finally, we intentionally restricted to England-based studies as differences in school systems may affect clustering. While estimates appear to be consistent across the different ages considered here and with values reported elsewhere, the extent to which these ICCs are generalisable to other countries should be explored further.

## Conclusions

Adequately powered studies are important to move forward the evidence for effective physical activity strategies. We have provided a comprehensive set of estimates of school-level ICCs and autocorrelations for accelerometer-measured physical activity in children and adolescents in England which can be used to plan new school-based cluster trials of physical activity interventions in schools. Estimates of the ICC were fairly consistent across a range of ages and settings, with estimates for weekday MVPA between 0.04 and 0.07 depending on age. It is important to use estimates appropriate to the study design, and that match the intended study population as closely as possible.

### Supplementary Information

Supplementary Material 1.

## Data Availability

The ICAD is open for data requests as a supported access resource. Information regarding the application process to access the data can be found at https://www.mrc-epid.cam.ac.uk/research/studies/icad/. Anonymised versions of the data from the Action 3:30 project have been deposited in the University of Bristol Research Data Repository (http://data.bris.ac.uk/data/). Data from the Active Lives Children and Young People Survey is available from the UK Data Service (SN 8854: https://doi.org/10.5255/UKDA-SN-8854–2). Data from B-Proact1v, AFLY5 and CHASE are available from the study investigators on reasonable request; see study citations for the relevant contact details.

## Abbreviations

AFLY5: Active for Life Year 5
ALCYP: Active Lives Children and Young People
ALSPAC: Avon Longitudinal Study of Parents and Children
CAC: Cluster autocorrelation
CHASE: Child Heart And health Study in England
ICAD: International Children’s Accelerometry Database
IAC: Individual autocorrelation
ICC: Intra-cluster correlation coefficient
MVPA: Moderate-to-vigorous physical activity
PEACH: Personal and Environmental Associations with Children's Health
RCT: Randomised controlled trial
SPEEDY: Sport, Physical Activity and Eating Behaviour: Environmental Determinants in Young people
