# Supplementary material for: School-level intra-cluster correlation coefficients and autocorrelations for children’s accelerometer-measured physical activity in England by age and gender
Source: BMC Med Res Methodol. 2024 Aug 9;24:179. doi: 10.1186/s12874-024-02290-7 (PMC11313128; doi:10.1186/s12874-024-02290-7)

# School-level intra-cluster correlation coefficients and autocorrelations for children’s accelerometer-measured physical activity in England by age and gender

**Authors**: Ruth Salway^*^, Russell Jago, Frank de Vocht, Danielle House_,_ Alice Porter, Robert Walker_,_ Ruth Kipping, Christopher G Owen, Mohammed T Hudda, Kate Northstone, Esther van Sluijs, On behalf of the International Children’s Accelerometry Database (ICAD) Collaborators

***Corresponding author:** Ruth Salway (ruth.salway@bristol.ac.uk)

**Table S1: Summary of accelerometer processing by study**

**Table S2: Missing accelerometer data by study**

**Table S3: Intra-cluster Correlation Coefficients (ICCs) with 95% confidence intervals (CIs) for boys’ MVPA and sedentary time by school year group**

**Table S4: Intra-cluster Correlation Coefficients (ICCs) with 95% confidence intervals (CIs) for girls’ MVPA and sedentary time by school year group.**

**Table S5: Total within-study variation for MVPA and sedentary time**

**Table S6: Intra-cluster Correlation Coefficients (ICCs) adjusted for baseline with 95% confidence intervals (CIs) for MVPA and sedentary time by school year group.**

**Table S7: Intra-cluster Correlation Coefficients (ICCs) with 95% confidence intervals (CIs) for self-reported MVPA by school year group.**

**Table S8: Intra-cluster correlation coefficients (ICCs) with 95% confidence intervals (CIs) for weekday MVPA for B-Proact1v Year 6 data only, processed using different criteria.**

**Table S9: Cluster autocorrelation (CAC) with 95% confidence intervals (CIs) for MVPA and sedentary time by length of follow-up.**

**Table S10: Individual autocorrelation (IAC) with 95% confidence intervals (CIs) for MVPA and sedentary time by length of follow-up.**

**Table S11: Primary schools: number of schools required to detect an increase of 5min weekday MVPA for different ICCs and designs**

**Table S12: Secondary schools: number of schools required to detect an increase of 5min weekday MVPA for different ICCs and designs**

**Figure S1: Intra-cluster correlation coefficient (ICC) and 95% confidence by age group and gender**

### Table S1: Summary of accelerometer processing by study

| Dataset | Accelerometer  model | Axis^1^ | No. days worn | MVPA  cut-point | Epochs | Wear time for valid day (min) |
| --- | --- | --- | --- | --- | --- | --- |
| ACTION 3:30 | Actigraph GT3X+ | vertical | 5 | 2295 | 10s | 500 |
| AFLY5 | Actigraph GT3X+ | vertical | 5 | 2295 | 10s | 480 |
| ALSPAC | Actigraph AM7164 | vertical | 7 | 2295 | 60s | 480 |
| B-Proact1v | Actigraph wGT3X-BT | vertical | 5 | 2295 | 60s | 480 |
| CHASE | Actigraph GT1M | vertical | 7 | 2000 | 5s | 600 |
| PEACH | Actigraph GT1M | vertical | 7 | 2295 | 60s | 480 |
| SPEEDY | Actigraph GT1M | vertical | 7 | 2295 | 60s | 480 |

^1^ Axis used for summary outcome count metric

**Table S2: Missing accelerometer data by study**

|  |  | Weekday | | Whole week | |
| --- | --- | --- | --- | --- | --- |
| Study | No. accelerometer measurements | No. valid measurements | % missing | No. valid measurements | % missing |
| B-Proact1v | 3604 | 3352 | 7% | 3454 | 4% |
| ACTION 3:30 | 938 | 844 | 10% | 888 | 5% |
| CHASE | 2035 | 1892 | 7% | 1934 | 5% |
| AFLY5 | 3629 | 2897 | 20% | 3110 | 14% |
| ALSPAC | 7014 | 6707 | 4% | 6832 | 3% |
| PEACH | 2532 | 2419 | 4% | 2441 | 4% |
| SPEEDY | 3026 | 2965 | 2% | 2990 | 1% |
|  | 22778 | 21076 | 7% | 21649 | 5% |

**Table S3: Intra-cluster Correlation Coefficients (ICCs) with 95% confidence intervals (CIs) for boys’ MVPA and sedentary time by school year group**

|  | Weekday | | Whole week | |
| --- | --- | --- | --- | --- |
|  | ICC | 95% CI | ICC | 95% CI |
| MVPA |  |  |  |  |
| Year 1 & 4 | 0.087 | (0.056, 0.133) | 0.091 | (0.060, 0.135) |
| Year 5 | 0.085 | (0.059, 0.121) | 0.052 | (0.033, 0.083) |
| Year 6 | 0.121 | (0.084, 0.171) | 0.101 | (0.069, 0.144) |
| Year 7 | 0.017 | (0.005, 0.058) | 0.008 | (0.001, 0.055) |
| Year 8 & 9 | 0.041 | (0.021, 0.080) | 0.027 | (0.012, 0.061) |
| Year 10 & 11 | 0.030 | (0.011, 0.079) | 0.022 | (0.006, 0.072) |
| Sedentary time |  |  |  |  |
| Year 1 & 4 | 0.026 | (0.011, 0.060) | 0.026 | (0.011, 0.061) |
| Year 5 | 0.038 | (0.017, 0.080) | 0.029 | (0.013, 0.064) |
| Year 6 | 0.060 | (0.029, 0.123) | 0.052 | (0.024, 0.107) |
| Year 7 | 0.003 | (0.000, 0.038) | 0.003 | (0.000, 0.030) |
| Year 8 & 9 | 0.015 | (0.005, 0.043) | 0.013 | (0.004, 0.039) |
| Year 10 & 11 | 0.002 | (0.000, 0.419) | 0.003 | (0.000, 0.171) |

MVPA=moderate to vigorous physical activity

**Table S4: Intra-cluster Correlation Coefficients (ICCs) with 95% confidence intervals (CIs) for girls’ MVPA and sedentary time by school year group**

|  | Weekday | | Whole week | |
| --- | --- | --- | --- | --- |
|  | ICC | 95% CI | ICC | 95% CI |
| MVPA |  |  |  |  |
| Year 1 & 4 | 0.084 | (0.054, 0.130) | 0.059 | (0.036, 0.096) |
| Year 5 | 0.090 | (0.062, 0.128) | 0.075 | (0.051, 0.109) |
| Year 6 | 0.100 | (0.070, 0.142) | 0.083 | (0.056, 0.120) |
| Year 7 | 0.066 | (0.040, 0.107) | 0.046 | (0.026, 0.082) |
| Year 8 & 9 | 0.095 | (0.060, 0.146) | 0.085 | (0.053, 0.134) |
| Year 10 & 11 | 0.084 | (0.048, 0.142) | 0.067 | (0.037, 0.117) |
| Sedentary time |  |  |  |  |
| Year 1 & 4 | 0.028 | (0.012, 0.065) | 0.020 | (0.008, 0.049) |
| Year 5 | 0.027 | (0.012, 0.061) | 0.019 | (0.008, 0.045) |
| Year 6 | 0.054 | (0.025, 0.112) | 0.040 | (0.018, 0.085) |
| Year 7 | 0.009 | (0.003, 0.029) | 0.010 | (0.003, 0.030) |
| Year 8 & 9 | 0.002 | (0.000, 0.030) | 0.001 | (0.000, 0.130) |
| Year 10 & 11 | 0.005 | (0.001, 0.032) | 0.002 | (0.000, 0.052) |

MVPA=moderate to vigorous physical activity

**Table S5: Total within-study variation for MVPA and sedentary time**

|  | Main model | | | | | | Adjusted for baseline | |
| --- | --- | --- | --- | --- | --- | --- | --- | --- |
|  | All | | Boys | | Girls | | All | |
|  | Variance | SD | Variance | SD | Variance | SD | Variance | SD |
| Weekday MVPA | | | | | | | | |
| Primary | 540.1 | 23.2 | 600.0 | 24.5 | 355.3 | 18.8 | 416.5 | 20.4 |
| Secondary | 745.7 | 27.5 | 877.0 | 29.6 | 517.4 | 22.7 | 584.6 | 24.2 |
| Whole week MVPA | | | | | | | | |
| Primary | 510.4 | 22.6 | 578.2 | 24.0 | 329.5 | 18.2 | 368.6 | 19.2 |
| Secondary | 660.3 | 25.7 | 778.2 | 27.9 | 438.2 | 20.9 | 504.3 | 22.5 |
| Weekday sedentary time | | | | | | | | |
| Primary | 2946.9 | 54.3 | 3045.3 | 55.2 | 2697.5 | 51.9 | 2796.0 | 52.9 |
| Secondary | 4887.1 | 69.9 | 5181.8 | 72.0 | 4041.3 | 63.6 | 4024.4 | 63.4 |
| Whole week sedentary time | | | | | | | | |
| Primary | 2727.1 | 52.2 | 2863.5 | 53.5 | 2492.2 | 49.9 | 2644.6 | 51.4 |
| Secondary | 4457.5 | 66.8 | 4809.7 | 69.4 | 3746.5 | 61.2 | 3748.9 | 61.2 |

MVPA=moderate-to-vigorous physical activity; SD=standard deviation

|  |  |  |  |
| --- | --- | --- | --- |

**Table S6: Intra-cluster Correlation Coefficients (ICCs) adjusted for baseline with 95% confidence intervals (CIs) for MVPA and sedentary time by school year group.**

|  | Weekday | | Whole week | |
| --- | --- | --- | --- | --- |
|  | ICC | 95% CI | ICC | 95% CI |
| MVPA |  |  |  |  |
| Primary | 0.062 | (0.041, 0.092) | 0.053 | (0.035, 0.080) |
| Secondary | 0.040 | (0.026, 0.062) | 0.037 | (0.023, 0.058) |
| Sedentary time |  |  |  |  |
| Primary | 0.061 | (0.036, 0.101) | 0.040 | (0.022, 0.071) |
| Secondary | 0.008 | (0.003, 0.021) | 0.007 | (0.003, 0.019) |

MVPA=moderate to vigorous physical activity

**Table S7: Intra-cluster Correlation Coefficients (ICCs) with 95% confidence intervals (CIs) for self-reported MVPA by school year group.**

|  | Weekday | | Whole week | |
| --- | --- | --- | --- | --- |
|  | ICC | 95% CI | ICC | 95% CI |
| All |  |  |  |  |
| Year 3 &4 | 0.087 | (0.077, 0.099) | 0.088 | (0.078, 0.100) |
| Year 5 & 6 | 0.072 | (0.063, 0.082) | 0.072 | (0.063, 0.082) |
| Year 7 & 8 | 0.063 | (0.054, 0.073) | 0.058 | (0.049, 0.067) |
| Year 9 & 11 | 0.040 | (0.033, 0.047) | 0.035 | (0.029, 0.042) |
| Boys |  |  |  |  |
| Year 3 &4 | 0.098 | (0.084, 0.115) | 0.102 | (0.087, 0.118) |
| Year 5 & 6 | 0.082 | (0.069, 0.096) | 0.082 | (0.069, 0.096) |
| Year 7 & 8 | 0.077 | (0.064, 0.093) | 0.070 | (0.058, 0.085) |
| Year 9 & 11 | 0.035 | (0.027, 0.046) | 0.031 | (0.024, 0.041) |
| Girls |  |  |  |  |
| Year 3 &4 | 0.087 | (0.074, 0.103) | 0.088 | (0.074, 0.104) |
| Year 5 & 6 | 0.084 | (0.072, 0.099) | 0.087 | (0.074, 0.101) |
| Year 7 & 8 | 0.074 | (0.061, 0.089) | 0.066 | (0.054, 0.081) |
| Year 9 & 11 | 0.046 | (0.037, 0.057) | 0.041 | (0.032, 0.051) |

MVPA=moderate to vigorous physical activity

**Table S8: Intra-cluster correlation coefficients (ICCs) with 95% confidence intervals (CIs) for weekday MVPA for B-Proact1v Year 6 data only, processed using different criteria.**

|  | ICC | 95% CI |
| --- | --- | --- |
| Main analysis processing^1^ | 0.137 | (0.085, 0.212) |
| MVPA cut-point: 2000^2^ | 0.147 | (0.093, 0.230) |
| Resolution: 5s epochs^2^ | 0.141 | (0.089, 0.220) |
| Resolution: 10s epochs^2^ | 0.141 | (0.089, 0.217) |
| Valid day: at least 300min^2^ | 0.135 | (0.084, 0.209) |
| Valid day: at least 600min^2^ | 0.127 | (0.077, 0.202) |
| Minimum valid days^2^: 1 | 0.129 | (0.080, 0.202) |
| Minimum valid days^2^: 3 | 0.140 | (0.085, 0.221) |

MVPA=moderate to vigorous physical activity.

^1^ Main analysis processing: Evenson cut-points (MVPA: 2295), 60s epochs, a valid day of at least 480min and a minimum of two valid days.

^2^ All other criteria as for main analysis.

**Table S9: Cluster autocorrelation (CAC) with 95% confidence intervals (CIs) for MVPA and sedentary time by length of follow-up.**

|  |  | Weekday | | Whole week | |
| --- | --- | --- | --- | --- | --- |
| Length of  follow-up | No. studies | CAC | 95% CI | CAC | 95% CI |
| MVPA |  |  |  |  |  |
| <1 year | 1 | 0.240 | (-0.459, 0.756) | 0.394 | (-0.314, 0.820) |
| 1 year | 3 | 0.698 | (0.567, 0.794) | 0.670 | (0.559, 0.758) |
| 2 years | 3 | 0.577 | (0.092, 0.841) | 0.461 | (-0.008, 0.765) |
| 3 years | 2 | 0.395 | (0.125, 0.611) | 0.500 | (0.257, 0.684) |
| 4-5 years | 3 | 0.561 | (0.089, 0.828) | 0.478 | (0.067, 0.751) |
| 1-5 years combined | 11 | 0.599 | (0.441, 0.721) | 0.550 | (0.411, 0.663) |
| Sedentary time |  |  |  |  |  |
| <1 year | 1 | 0.459 | (-0.240, 0.845) | 0.509 | (-0.178, 0.862) |
| 1 year | 3 | 0.520 | (0.377, 0.639) | 0.546 | (0.409, 0.660) |
| 2 years | 3 | 0.435 | (-0.261, 0.833) | 0.450 | (-0.194, 0.823) |
| 3 years | 2 | 0.205 | (-0.083, 0.462) | 0.427 | (0.171, 0.629) |
| 4-5 years | 3 | 0.439 | (-0.060, 0.762) | 0.327 | (0.124, 0.503) |
| 1-5 years combined | 11 | 0.429 | (0.240, 0.589) | 0.465 | (0.298, 0.605) |

**Table S10: Individual autocorrelation (IAC) with 95% confidence intervals (CIs) for MVPA and sedentary time by length of follow-up.**

|  |  | Weekday | | Whole week | |
| --- | --- | --- | --- | --- | --- |
| Length of  follow-up | No. studies | IAC | 95% CI | IAC | 95% CI |
| MVPA |  |  |  |  |  |
| <1 year | 1 | 0.435 | (0.309, 0.546) | 0.542 | (0.437, 0.632) |
| 1 year | 5 | 0.516 | (0.467, 0.561) | 0.540 | (0.475, 0.600) |
| 2 years | 3 | 0.477 | (0.429, 0.522) | 0.475 | (0.425, 0.522) |
| 3 years | 3 | 0.453 | (0.404, 0.501) | 0.443 | (0.382, 0.501) |
| 4 years | 3 | 0.388 | (0.343, 0.431) | 0.377 | (0.309, 0.441) |
| 5 years | 2 | 0.343 | (0.176, 0.492) | 0.350 | (0.164, 0.512) |
| 1-5 years combined | 16 | 0.459 | (0.423, 0.494) | 0.461 | (0.414, 0.504) |
| Sedentary time |  |  |  |  |  |
| <1 year | 1 | 0.410 | (0.281, 0.524) | 0.371 | (0.254, 0.477) |
| 1 year | 5 | 0.399 | (0.280, 0.505) | 0.394 | (0.271, 0.505) |
| 2 years | 3 | 0.392 | (0.275, 0.498) | 0.373 | (0.220, 0.509) |
| 3 years | 3 | 0.332 | (0.265, 0.394) | 0.353 | (0.248, 0.450) |
| 4 years | 3 | 0.356 | (0.310, 0.399) | 0.371 | (0.318, 0.423) |
| 5 years | 2 | 0.283 | (0.216, 0.347) | 0.268 | (0.204, 0.330) |
| 1-5 years combined | 16 | 0.367 | (0.320, 0.411) | 0.364 | (0.313, 0.414) |

**Table S11: Primary schools: number of schools required to detect an increase of 5min weekday MVPA for different ICCs and designs**

|  |  |  | ICC | |  |  |  |
| --- | --- | --- | --- | --- | --- | --- | --- |
|  |  |  | 0.02 | 0.04 | | 0.06 | 0.08 |
| **25 pupils per school** | |  |  |  | |  |  |
| **Cluster RCT** | |  |  |  | |  |  |
|  | Two-arm |  | 44 | 56 | | 68 | 82 |
|  | adjusted for baseline |  | 34 | 44 | | 54 | 64 |
| **Stepped wedge (same schools): 2 steps** | |  |  |  | |  |  |
|  | Cross-sectional |  | 38 | 46 | | 54 | 62 |
|  | Cohort |  | 26 | 34 | | 40 | 48 |
| **Stepped wedge (same schools): 3 steps** | |  |  |  | |  |  |
|  | Cross-sectional |  | 24 | 30 | | 33 | 39 |
|  | Cohort |  | 18 | 21 | | 24 | 30 |
| **50 pupils per school** | |  |  |  | |  |  |
| **Cluster RCT** | |  |  |  | |  |  |
|  | Two-arm |  | 30 | 44 | | 56 | 70 |
|  | adjusted for baseline |  | 24 | 34 | | 44 | 54 |
| **Stepped wedge (same schools): 2 steps** | |  |  |  | |  |  |
|  | Cross-sectional |  | 24 | 32 | | 40 | 46 |
|  | Cohort |  | 18 | 24 | | 32 | 40 |
| **Stepped wedge (same schools): 3 steps** | |  |  |  | |  |  |
|  | Cross-sectional |  | 15 | 21 | | 24 | 30 |
|  | Cohort |  | 12 | 15 | | 21 | 24 |

80% power; 5% significance level;

total standard deviation=20.4 min (adjusted for baseline) 23.2 min (all other designs)

stepped wedge designs: CAC=0.6; IAC =0.5 (cohort only)

**Table S12: Secondary schools: number of schools required to detect an increase of 5min weekday MVPA for different ICCs and designs**

|  |  |  | ICC | |  |  |  |
| --- | --- | --- | --- | --- | --- | --- | --- |
|  |  |  | 0.02 | 0.04 | | 0.06 | 0.08 |
| **25 pupils per school** | |  |  |  | |  |  |
| **Cluster RCT** | |  |  |  | |  |  |
|  | Two-arm |  | 60 | 78 | | 96 | 114 |
|  | adjusted for baseline |  | 46 | 60 | | 74 | 88 |
| **Stepped wedge (same schools): 2 steps** | |  |  |  | |  |  |
|  | Cross-sectional |  | 54 | 64 | | 76 | 86 |
|  | Cohort |  | 36 | 46 | | 56 | 66 |
| **Stepped wedge (same schools): 3 steps** | |  |  |  | |  |  |
|  | Cross-sectional |  | 33 | 39 | | 45 | 51 |
|  | Cohort |  | 24 | 30 | | 36 | 42 |
| **50 pupils per school** | |  |  |  | |  |  |
| **Cluster RCT** | |  |  |  | |  |  |
|  | Two-arm |  | 40 | 60 | | 78 | 96 |
|  | adjusted for baseline |  | 32 | 46 | | 60 | 76 |
| **Stepped wedge (same schools): 2 steps** | |  |  |  | |  |  |
|  | Cross-sectional |  | 34 | 44 | | 54 | 64 |
|  | Cohort |  | 24 | 34 | | 44 | 54 |
| **Stepped wedge (same schools): 3 steps** | |  |  |  | |  |  |
|  | Cross-sectional |  | 21 | 27 | | 33 | 39 |
|  | Cohort |  | 15 | 21 | | 27 | 33 |
| **75 pupils per school** | |  |  |  | |  |  |
| **Cluster RCT** | |  |  |  | |  |  |
|  | Two-arm |  | 34 | 54 | | 72 | 90 |
|  | adjusted for baseline |  | 28 | 42 | | 56 | 70 |
| **Stepped wedge (same schools): 2 steps** | |  |  |  | |  |  |
|  | Cross-sectional |  | 26 | 36 | | 48 | 58 |
|  | Cohort |  | 20 | 30 | | 40 | 50 |
| **Stepped wedge (same schools): 3 steps** | |  |  |  | |  |  |
|  | Cross-sectional |  | 18 | 24 | | 30 | 36 |
|  | Cohort |  | 12 | 18 | | 24 | 30 |

80% power; 5% significance level;

total standard deviation=24.2 min (adjusted for baseline) 27.5 min (all other designs)

stepped wedge designs: CAC=0.6; IAC =0.5 (cohort only)

**Figure S1: Intra-cluster correlation coefficient (ICC) and 95% confidence by age group and gender**


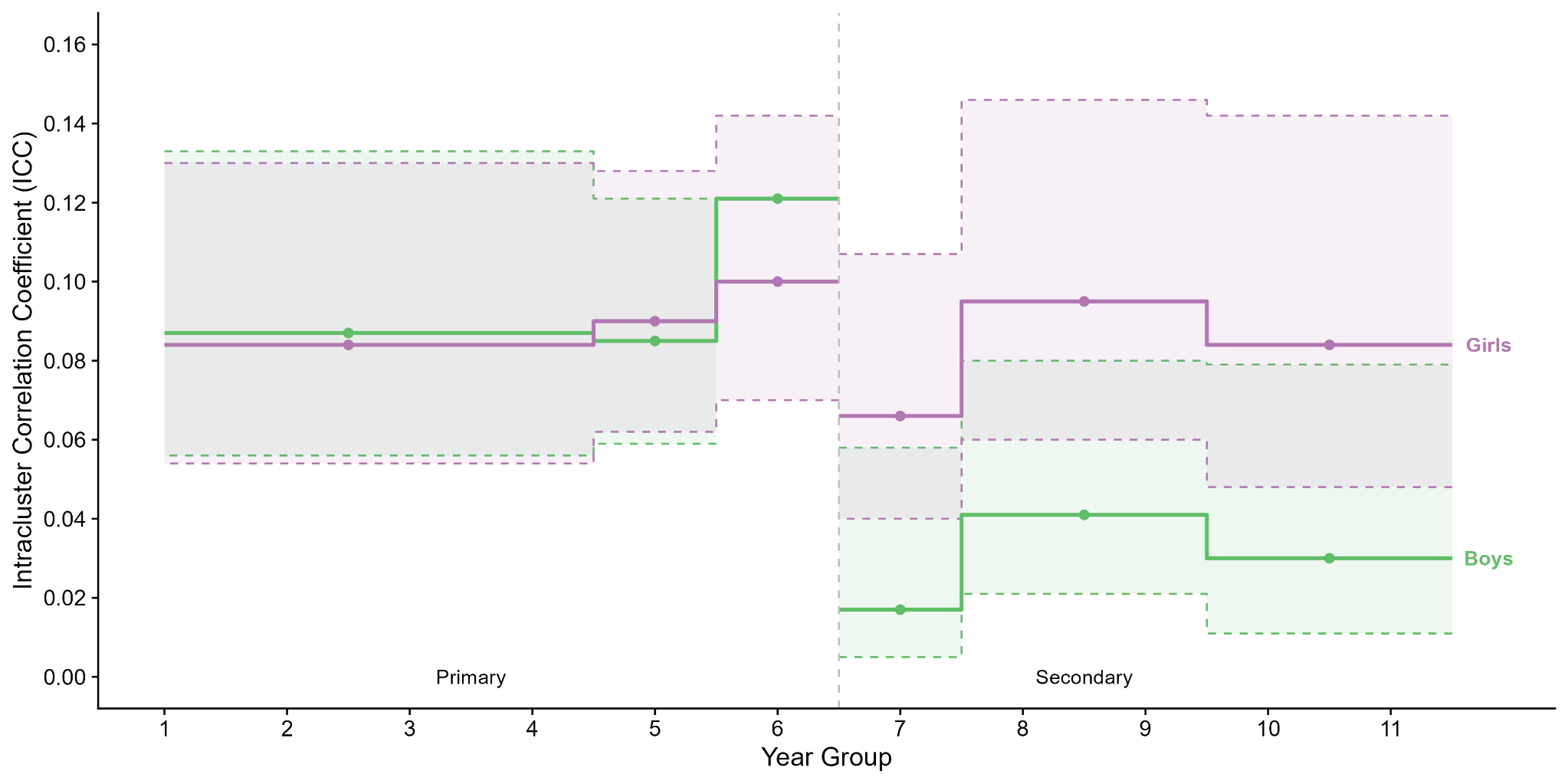

Supplement: Supplementary file 1 — Supplementary Material 1. [file 12874_2024_2290_MOESM1_ESM.docx]
